# Cause of death in patients with poststroke epilepsy: Results from a nationwide cohort study

**DOI:** 10.1371/journal.pone.0174659

**Published:** 2017-04-05

**Authors:** Julia Hansen, Signild Åsberg, Eva Kumlien, Johan Zelano

**Affiliations:** 1 Department of neurology, Sahlgrenska university hospital and Sahlgrenska Academy, University of Gothenburg, Gothenburg, Sweden; 2 Department of Medical Sciences, Uppsala University, Uppsala, Sweden; 3 Department of Neuroscience, Uppsala University, Uppsala, Sweden; Medizinische Universitat Innsbruck, AUSTRIA

## Abstract

The risk of death is increased for persons with epilepsy. The literature on causes of death in epilepsy is based mainly on cohorts with epilepsy of mixed aetiologies. For clinical purposes and improved understanding of mortality in different epilepsies, more information is needed on mortality in epilepsies of specific causes. In poststroke epilepsy (PSE), seizures occur in a setting of vascular disease and high mortality rates. The extent to which epilepsy contributes to mortality in this patient group is poorly understood. We therefore aimed to describe causes of death (COD) in PSE on a national scale. A previously identified cohort of 7740 patients with epilepsy or seizures after a stroke in 2005–2010 was investigated. A total of 4167 deaths occurred before the end of 2014. The standardized mortality ratio for the study cohort was 3.56 (95% CI: 3.45–3.67). The main underlying causes of death were disorders of the circulatory system (60%) followed by neoplasms (12%). Diseases of the nervous system were the sixth leading underlying COD (3%), and epilepsy or status epilepticus was considered the underlying COD in approximately a similar proportion of cases as neurodegenerative disorders (0.9% and 1.1%, respectively). Epilepsy was considered a contributing COD in 14% of cases. Our findings highlight the importance of optimal management of vascular morbidity in patients with PSE. The large proportion of patients with epilepsy as a contributing COD indicate the need of high ambitions also regarding the management of seizures in patients with PSE.

## Introduction

Persons with epilepsy have an increased risk of death compared to the standard population; previous work has demonstrated that the standardized mortality ratio (SMR) for patients with epilepsy in general is 1.5–3 [1, 2]. The cause of death (COD) is often linked to the underlying aetiology of epilepsy, and more so if death occurs within the first few years of the epilepsy diagnosis [3]. The current literature is most often based on relatively small cohorts with rather few deaths occurring in each aetiological category [3–7]. Exceptions exist, but the focus of these larger studies was mortality associated with epilepsy in general [8, 9]. Such studies are of great importance for an understanding of the burden of epilepsy, proper resource allocation in healthcare, and public health initiatives. However, the epilepsy classifications often used (for instance “remote symptomatic”) are too broad to easily inform clinical practice and pathophysiological understanding. A need for larger studies on epilepsies of different causes has been identified in a recent review[2].

Cerebrovascular disease is the most common cause of epilepsy after middle age, and poststroke epilepsy (PSE) complicates 6–8% of stroke according to fairly large and well-designed contemporary investigations [10, 11]. We have recently undertaken a nationwide registry-based cohort study on PSE in Sweden, and identified 7740 patients that suffered a first epilepsy or seizure diagnosis following a stroke in 2005–2010, resulting in a cumulative incidence of 7,3% [12]. Mortality in the cohort was high, and PSE was associated with an increased hazard ratio of 1.36 (95% CI 1.20–1.55) for death after adjustments for stroke severity. In order to improve survival for patients with PSE, a better understanding of COD is required. Since PSE occurs in a setting of vascular disease and seizures often are focal and non-life threatening, it is likely that COD data from general epilepsy populations are not readily generalizable to PSE. We therefore wanted to investigate COD in patients with PSE.

Some data exist. In a recent UK community-based cohort study noncerebral neoplasms, cardiovascular disease, and cerebrovascular disease were the most common COD, accounting for 59% of fatalities, as assessed by proportionate mortality ratios [3]. Epilepsy was the underlying COD in 3% of cases. Other studies have demonstrated high risks of dying in cerebrovascular disease for patients with epilepsy in general [8]. Based on these findings, vascular disease is likely to be a major COD among PSE patients. However, the contribution of epilepsy to mortality in PSE is not known. Similarly, there is limited knowledge on what proportion of deaths in patients with PSE result from external causes that are increased in general epilepsy populations, like suicide, transport accidents or drowning [2].

In the present study, we aimed to describe causes of deaths in a previously identified cohort of 7740 patients that developed epilepsy following a stroke in 2005–2010.

## Materials and methods

### Study population

**Fig 1:**
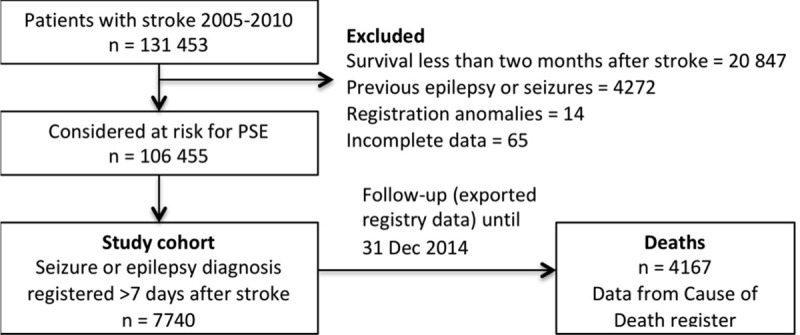
Flow chart. Flow chart describing how the cohort was identified.

An incident cohort with a first ever seizure, status epilepticus or epilepsy diagnosis following stroke in 2005–2010 was identified through cross-referencing of the Swedish Stroke Register and the National Patient Register (NPR) and Cause of Death (COD) Register, as previously described [12]. Patients with epilepsy, seizure or status epilepticus diagnosis prior to the stroke were excluded, as were patients that died within two months of the index stroke in order to reduce the influence of acute case fatality. The study years were chosen to reflect current practice, but also allow development of PSE and sufficient follow-up time. From a total of 106455 stroke patients fulfilling these criteria, 7740 patients had at least one seizure, epilepsy or status epilepticus diagnosis, more than one week after the index stroke, until the end of 2014. The average follow-up time was 4.8 years (SD 2.7 years). Inclusion is described in Fig 1.

### Registers and classification of COD

The NPR and COD registers are nation-wide, with mandatory annual reporting from all healthcare providers in Sweden. The NPR was originally a hospital discharge register with complete coverage from 1987, and from 2001 hospital-based outpatient care is also reported. Diagnoses are registered for ICD-10 codes. For the purpose of this study, patients with occurrence of any of ICD-10-codes G40, G41, or R56.8 more than 7 days after the stroke index date were classified as PSE. The positive predictive value of the epilepsy diagnosis in the NPR is not known, but the register is frequently used to identify epilepsy in register-based research [13, 14]. Causes of death in Sweden are reported in text on a death certificate by a physician and coded as International Classification of Disease (ICD)-10 codes by the National Board of Health and Welfare according to the instruction in the ICD-10 manual. Missing death certificates (0.8%), insufficient information on death certificates (2.7%) and coding errors (estimated at 1.8%) are potential sources of error, as well as the low current autopsy rate in Sweden, which is less than 20% [15]. External validation of the COD regarding stroke in the general population found misclassification in 14% [16]. In the present study, COD was classified according to the ICD-10-code (G40 for epilepsy, G41 for status epilepticus, and R568 for seizure), and grouped according to ICD-10 chapter.

Linking of registers was performed using the unique personal identification number of every Swedish citizen. The National Board of Health and Welfare anonymised all data after linkage and before we were given access to them. The study was performed in agreement with privacy legislation in Sweden and approved by the regional ethics committee in Gothenburg (approval number 187–15). A STROBE checklist for cohort studies was completed.

### Statistical methods

SMR was calculated as the ratio between deaths that occurred and the expected number of deaths, based on the data for age and gender stratified mortality rates in Sweden for the study years. COD are presented as observed cases and proportionate mortality ratio (the ratio between a COD and all deaths in the population). Confidence intervals for proportions with more than 5 observations were calculated for a 95% level of confidence using the adjusted Wald method in Excel and online tools at www.openepi.com for SMR.

## Results

**Fig 2:**
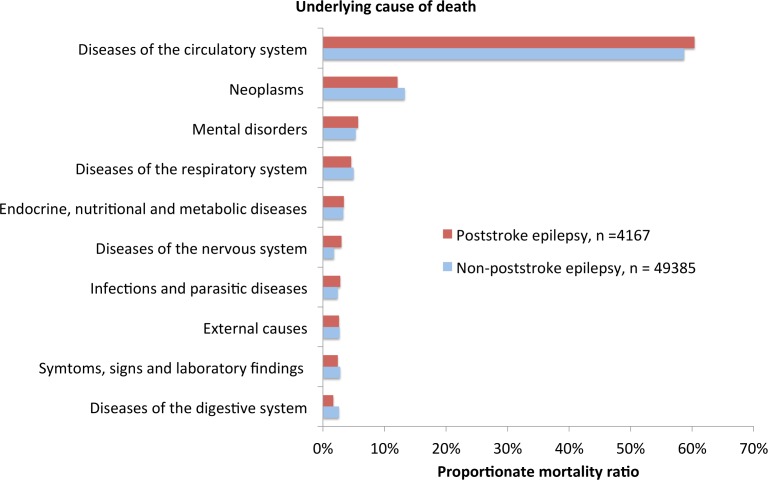
Proportionate mortality ratios. Proportionate mortality ratios for the ten most common COD for patients with PSE (number of deaths = 4167). PMR for patients that suffered stroke but did not develop PSE are also shown (number of deaths = 49385).

The characteristics of the study population are presented in Table 1. A total of 4167 deaths occurred until the end of 2014, resulting in a SMR of 3.56 (95% CI: 3.45–3.67). We first assessed underlying COD (Fig 2, S1 Table). The top five diagnostic groups were diseases of the circulatory system (60%, 95%CI: 58.9–61.9), neoplasms (12%, 95%CI: 11.2–13.2), mental disorders (5.7%, 95%CI: 5.1–6.5), disorders of the respiratory system (4.6%, 95%CI: 4.0–5.2) and endocrine, nutritional and metabolic disorders (3.4%, 95%CI: 2.9–3.5). Mental disorders include dementias. ICD-10 codes for Alzheimers disease, vascular dementia, dementia in other diseases, and unspecified dementia (F00-F03) accounted for 96% of this category (5.5%, 95%CI: 4.8–6.2). As a reference, we also assessed the PMR for the total of 49385 deaths that occurred in patients that suffered stroke during the study period, but had not developed PSE.

**Table 1:** Study cohort.

Study cohort, n = 7740			
**Age at diagnosis and time to PSE**		**years**	
Age at index stroke (years)	Mean (range)	72 (14–99)	
	Median	74	
Time stroke to epilepsy (years)	Mean (range)	1.87 (0.02–9.8)	
	Median	1.11	
**Clinical characteristics**		N	%
Age at stroke (“young stroke)	<45 yrs	239	3.1%
	> = 45 yrs	7501	96.9%
Sex	Male	4261	55.1%
	Female	3479	44.9%
Stroke previously		1473	19.2%
Stroke type	ICH	1263	16.3%
	Infarction	6477	83.7%
Thrombolysis		513	6.7%
Single seizure		1195	15.4%
Single status epilepticus		129	1.7%
Deaths before 31/12 2014		4167	53.8%

The study cohort consisted of a total of 7740 patients with a diagnosis of epilepsy, seizure or status epilepticus more than one week after stroke in 2005–2010.

We next analysed the two most common COD and COD relating to the nervous system in detail. Among diseases of the circulatory system, cerebrovascular diseases were the most common causes of death (31%), followed by ischemic heart diseases (14%) and other forms of heart disease (11%, Table 2). Malignant neoplasms accounted for 11% of deaths. Within diseases of the nervous system, degenerative disorders (1.1%) accounted for approximately the same number of deaths as episodic and paroxysmal disorders (1%, most of which were epilepsy-related).

**Table 2:** Common underlying causes of death.

Underlying COD		n	PMR (95% CI)
**Diseases of the circulatory system**		**2517**	**60 (58.9–61.9) %**
**Cerebrovascular diseases**		1308	31(30.0–32.8)%
	*Sequelae of cerebrovascular disease*	*535*	*13 (11*.*9–13*.*9)%*
	*Stroke*, *not specified type*	*323*	*7*.*8 (7*.*0–8*.*6)%*
	*Cerebral infarction*	*215*	*5*.*2 (4*.*5–5*.*9)%*
	*Intracerebral haemorrhage*	*88*	*2*.*1(1*.*7–2*.*6)%*
**Ischemic heart disease**		583	14 (13.0–15.1)%
	*Chronic ischemic heart disease*	*327*	*7*.*8 (7*.*1–8*.*7)%*
	*Acute myocardial infarction*	*240*	*5*.*8 (5*.*1–6*.*5)%*
**Other forms of heart disease**		462	11 (10.2–12.1)%
**Neoplasms**		**505**	**12 (11.2–13.2)%**
**Malignant neoplasms**		463	11 (10.2–12.1)%
**Neoplasms of uncertain and unknown behaviour**		37	0.9 (0.6–1.2)%
**Benign neoplasms**		5	0.1%
**Diseases of the nervous system**		**123**	**3.0 (2.5–3.5) %**
**Other degenerative diseases of the nervous system**		45	1.1 (0.8–1.5)%
**Episodic and paroxysmal disorders**		40	1.0 (0.7–1.3)%
**Extrapyramidal and movement disorders**		11	0.3 (0.1–0.5)%

The three most common underlying causes of deaths within the circulatory system, neoplasms and the nervous system, with specified diagnoses for common circulatory disorders.

**Fig 3:**
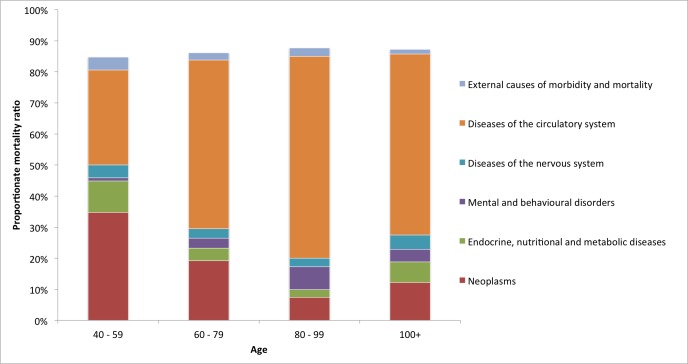
Proportionate mortality ratios for age groups. Proportionate mortality ratios for underlying causes of death per age group. The number of deaths was 98 for age 40–59, 1352 for age 60–79, 2517 for age 80–99, and 196 for age 100+. The remaining 4 deaths occurred in ages 20–39, PMR is not shown.

The underlying COD was also analysed depending on type of index stroke, age at death, and time from epilepsy onset. In comparison with causes of death after ischemic stroke, neoplasms were more common in cases of intracerebral haemorrhage (ICH, PMR 88 / 509 = 17.3% vs. 417 / 3658 = 11.4%, p < 0.05), but the ranking of the top five COD-groups were the same in cases of IS and ICH. Diseases of the circulatory system was coded as the underlying COD in 272 / 509 = 53.4% and 2245 / 3658 = 61.4% of cases of ICH and IS, respectively (p < 0.05) Brain tumour diagnoses (ICD-10-codes D43 or C71) were significantly more common as underlying causes of death in patients with ICH than IS (20/509 = 3.9% vs. 65/3658 = 1.8%, p < 0.05). For younger age groups (age 40–59), the proportion of deaths due to circulatory system diseases was lower compared with higher age groups (age 60+), but the total number of deaths (n = 98) was also much smaller (Fig 3, S2 Table).

**Fig 4:**
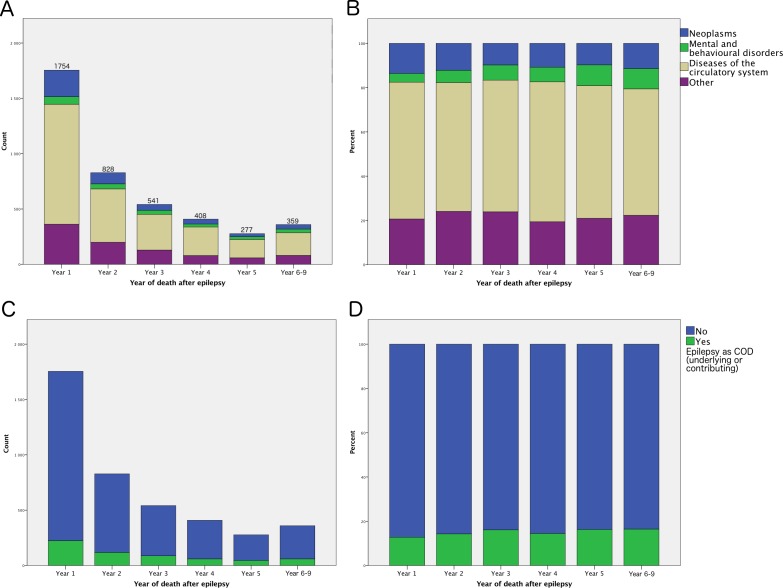
Causes of death stratified by time of death. Top row: Absolute counts (A) and proportions (B) of the three largest categories of COD. Lower row: Absolute count (C) and proportion (D) of patients with epilepsy (ICD-10 code G40) coded as an underlying or contributing COD.

We also analysed COD over time. The proportions of cerebrovascular diseases and neoplasms were relatively constant over the observed time, as was the proportion of patients with a diagnosis of epilepsy (G40) coded as underlying or contributing COD (Fig 4).

We then analysed epilepsy-related COD; epilepsy was coded as underlying COD in 24 cases, and status epilepticus was coded as the underlying COD in 15 cases (Table 3). Epilepsy was coded as a contributing COD in 592 cases (these included the 24 deaths where epilepsy was given as the underlying COD). Epilepsy, status epilepticus or seizures were noted as a contributing COD in 695 cases, representing 17% of all deaths in the cohort (Table 3). We also investigated the underlying COD in patients where epilepsy was coded as a contributing cause. Also among these patients, circulatory disorders consisted the underlying cause in a majority of deaths (Table 4). Death from external cause that are increased in general epilepsy populations, like suicide, transport accidents or drowning, were seen in very small numbers (S3 Table)

**Table 3:** Death from epilepsy.

	n	PMR (95%CI)
**Underlying COD**		
**Epilepsy**	24	0.6 (0.4–0.9)%
**Status epilepticus**	15	0.4 (0.2–0.6)%
**Epilepsy or status epilepticus**	39	0.9 (0.7–1.3)%
**Underlying and/or contributing COD**		
**Epilepsy**	592	14 (13.2–15.3)%
**Epilepsy, status epilepticus or seizure**	695	17 (15.6–17.8)%

The number of cases with epilepsy, status epilepticus, or seizures listed as a COD, either underlying or contributing.

**Table 4:** Underlying COD in patients with epilepsy as a contributing cause.

Diagnostic chapter	n	PMR (95%CI)
**Diseases of the circulatory system (9)**	394	67 (62.7–70.3)%
**Diseases of the nervous system (6)**	43	7.3 (5.4–9.7)%
**Mental and behavioural disorders (5)**	39	6.6 (4.8–8.9)%
**Dementia (F00-F03)**	38	6.4 (4.7–8.7)%
**Neoplasms (2)**	36	6.1 (4.4–8.3)%
**Endocrine, nutritional and metabolic diseases (4)**	21	3.5 (2.3–5.4)%
**Certain infectious and parasitic diseases (1)**	21	3.5 (2.3–5.4)%
**Diseases of the respiratory system (10)**	15	2.5 (1.5–4.2)%
**External causes of morbidity and mortality (20)**	12	2.0 (1.1–3.6)%

The most common underlying COD in cases with epilepsy listed as contributing COD, n = 592. () indicate ICD-10 chapter number or ICD-10 codes for dementias.

## Discussion

We present data on causes of death from a large nationwide registry-based study on PSE. To our knowledge, this is the largest report on causes of death in PSE, and the results should be informative for patients and caregivers. The study design was pragmatic, which is associated with drawbacks discussed below. These are however unlikely to be of such magnitude that they influence the main findings.

The SMR for the study cohort was high, in line with other smaller studies on remote symptomatic epilepsy[2]. Our main findings is that the most common underlying COD in PSE were disease of the circulatory system and that epilepsy was considered an underlying COD in only 0.6% of cases. Naturally, findings in single studies should be interpreted cautiously but our findings indicate that COD are different in patients with PSE compared to patients with epilepsy in general. For instance, in a recent community-based study, epilepsy was the underlying cause in 3% of deaths [3]. Overall, our findings are in agreement with the present literature on epilepsy of remote symptomatic causes, which indicate that cerebrovascular disease is a common COD [2, 3, 8].

The PMRs for the most common categories of COD were similar in patients with and without PSE. Importantly, patients without PSE after stroke are not perfect comparators; patients with PSE have typically suffered a more severe or haemorrhagic stroke than patients that do not develop PSE[12]. Survival is also different, as is observation time given that PSE develops some time after stroke. For these reasons, most importantly that exposure (PSE) is related to an important prognostic factor (stroke severity), we decided against statistical comparisons. However, if these limitations are kept in mind, the comparison provides valuable information for patients with newly diagnosed PSE and their treating physicians. Judging by our data, epilepsy after stroke does not cause a major shift in the leading causes of death within the first years after stroke, in comparison to patients without epilepsy after stroke.

The COD of the 2.4% deaths that occurred in patients under the age of 60 were different from that seen in older patients, so our conclusions do not extend to this group. Stroke in younger age groups typically occur for different reasons, perhaps due to co-morbidities, and neoplasms were much more common as COD. Given the uncertain nature of death certificate data, extra caution is warranted when interpreting small numbers or proportions. There was a larger proportion of neoplasm as underlying COD in cases of ICH. One possible explanation could be that brain tumours are more likely to cause ICH than IS, or that patients with ICH that survive two months are less likely to die from circulatory diseases than patients with infarctions—making all other CODs more common. One should not infer causality from our observational data.

The clinical relevance of our findings is the importance of vascular disease in patients with PSE. Patients with epilepsy after stroke seem to die from vascular disease and vascular prophylaxis should be ambitious in this high-risk population. More research is also needed on possible negative interactions between epilepsy and vascular disease. Seizures may hamper rehabilitation efforts and enzyme-inducing antiepileptic drugs may interfere with cardiovascular secondary prophylaxis, or have detrimental vascular effects on their own.

Epilepsy was not a common underlying COD, accounting for only 0.6% of deaths—less than the 3–4% observed in general epilepsy populations [3, 17]. However, epilepsy was listed as a contributing COD in 14% of deaths, indicating that the impact of epilepsy on case-fatality is probably clinically relevant. Studies on death certificates have intrinsic limitations in that we cannot know the reason for listing epilepsy on the death certificate. Presumably, active epilepsy is more likely to be entered than a seizure disorder in remission. We have previously demonstrated in a small single-centre study (n = 35) that only 55% of patients with PSE had achieved seizure-freedom with the first or second AED at last follow-up[18]. This contrasts with the notion that PSE is easy to treat, and is in agreement with two other older real-life studies with seizure freedom rates of 54% (n = 36) and 67% (n = 46)[19–21]. These seizure freedom rates are not very impressive compared to other aetiologies of epilepsy. Worryingly, the real-life data contrast considerably with the excellent efficacy noted in some prospective evaluations of monotherapy [22–25] This discrepancy, taken together with our present finding that epilepsy was listed as a contributing COD in 14% of deaths in our cohort, indicate that higher ambitions are needed in treatment of seizures in PSE. Uncontrolled epilepsy may perhaps contribute to high vascular mortality, by hampering rehabilitation efforts.

Our study has limitations. The registers used have excellent coverage of 90–96% for the Swedish stroke register and mandatory reporting to the NPR and COD registers (less than 1% and 3% missing data, respectively). Nonetheless, misclassification and other registration errors occur. Death certificate studies in epilepsy may be associated with difficulties due to under- as well as overestimation of epilepsy as a COD depending on the knowledge of the certifying physician, autopsy rates, etc. This is a concern not only for registry-based investigations.

Furthermore, as the high risk of seizure recurrence following a remote seizure after stroke that is now encompassed in the ILAE definition of epilepsy was not well known during the study years, we used a wide definition of PSE and included all patients with a first code for epilepsy, single seizure, or status epilepticus occurring more than one week after the index stroke. Our definition may have led to inclusion of patients with epilepsy due to other remote symptomatic causes than cerebrovascular disease or acute symptomatic seizures due to subsequent strokes. The impact of such imperfections is however most likely small; more than 83% of the 7740 PSE patients were not included based on a single code for seizure or status epilepticus. In our opinion, our inclusion criteria is unlikely to influence our main finding regarding circulatory diseases being the most common COD, as illustrated by our separate analysis of underlying COD in patients with a code for epilepsy as a contributing COD (Table 4). The limitations are important, but complex issues like mortality in PSE needs to be examined by complementary methods. Large nationwide registry-based studies serve as a useful complement to smaller studies, which are often subject to more inclusion bias but have more detailed information as a result of access to individual medical records.

In summary, the most common COD was diseases of the circulatory system. Epilepsy was listed a contributing COD in a relatively high proportion of cases. Our findings demonstrate the importance of more research on management of PSE, especially on how neurologists should take into account and optimize treatment of vascular disease in addition to efforts aimed at seizure control.

## Supporting information

S1 Table Causes of death and proportionate mortality rate in all patients. (PDF)

S2 Table Underlying causes of death per age group at time of death. (PDF)

S3 Table Death from suicide, drowning and transport accidents in patients with PSE. (PDF)
