# Supplementary material for: Cause of death in patients with poststroke epilepsy: Results from a nationwide cohort study
Source: PLoS One. 2017 Apr 5;12(4):e0174659. doi: 10.1371/journal.pone.0174659 (PMC5381780; doi:10.1371/journal.pone.0174659)
Supplement: S1 Table — (PDF) [file pone.0174659.s001.pdf]

Table S1. Causes of death and proportionate mortality rate in all patients

Patients with poststroke epilepsy		**(deaths = 4167)**
**ICD-10 Chapter (abbreviated titles)**	**n**	**PMR (95%CI)**
Diseases of the circulatory system	2517	60.4% (58.9-61.9)
Neoplasms	505	12.1% (11.2-13.2)
Mental disorders	238	5.7% (5.1-6.5)
Diseases of the respiratory system	190	4.6% (4.0-5.2)
Endocrine, nutritional and metabolic diseases	141	3.4% (2.9-4.0)
Diseases of the nervous system	123	3.0% (2.5-3.5)
Infections and parasitic diseases	116	2.8% (2.3-3.3)
External causes	107	2.6% (2.1-3.1)
Symtoms, signs and laboratory findings	99	2.4% (2.0-2.9)
Diseases of the digestive system	72	1.7% (1.4-2.2)
Diseases of the genitourinary system	34	0.8% (0.6-1.1)
Diseases of the musculoskeletal system and connective tissue	14	0.3% (0.2-0.6)
Congenital malformations, chromosomal abnormalities	5	0.1%
Diseases of the blood and blood forming organs	4	0.1%
Diseases of the skin	2	0.0%
Pregnancy, childbirth, and puerperium	0	0.0%
