# Supplementary material for: Cause of death in patients with poststroke epilepsy: Results from a nationwide cohort study
Source: PLoS One. 2017 Apr 5;12(4):e0174659. doi: 10.1371/journal.pone.0174659 (PMC5381780; doi:10.1371/journal.pone.0174659)
Supplement: S2 Table — (PDF) [file pone.0174659.s002.pdf]

Table S2. Underlying causes of death per age group at time of death.

Age	0-19		20-39		40-59		60-79		80-99		100+	
**Chapter**	**n**	**%**	**n**	**%**	**n**	**%**	**n**	**%**	**n**	**%**	**n**	**%**
Certain infectious and parasitic diseases	0	0%	0	0%	3	3.1%	40	3.0%	69	2.7%	4	2.0%
Neoplasms	0	0%	1	25%	34	34.7%	259	19.2%	187	7.4%	24	12.2%
Diseases of the blood and blood-forming	0	0%	0	0%	0	0%	0	0.0%	4	0.2%	0	0%
Endocrine, nutritional and metabolic diseases	0	0%	0	0%	10	10.2%	54	4%	64	2.5%	13	6.6%
Mental and behavioral disorders	0	0%	0	0%	1	1%	44	3.3%	185	7.4%	8	4.1%
Diseases of the nervous system	0	0%	0	0%	4	4.1%	41	3%	69	2.7%	9	4.6%
Diseases of the circulatory system	0	0%	2	50%	30	30.6%	734	54.3%	1637	65.0%	114	58.2%
Diseases of the respiratory system	0	0%	0	0%	2	2%	68	5.0%	112	4.4%	8	4.1%
Diseases of the digestive system	0	0%	1	25%	3	3.1%	28	2.1%	39	1.5%	1	0.5%
Diseases of the skin and subcutaneous tissue	0	0%	0	0%	0	0%	0	0%	2	0,1%	0	0%
Diseases of the musculoskeletal system	0	0%	0	0%	0	0%	10	0,7%	4	0.2%	0	0%
Diseases of the genitourinary system	0	0%	0	0%	0	0%	15	1.1%	16	0.6%	3	1.5%
Congenital malformations , deformations and chromosomal abn	0	0%	0	0%	0	0%	5	0.4%	0	0%	0	0%
Symptoms, signs and abnormal clinical and laboratory findings	0	0%	0	0%	7	7.1%	23	1.7%	60	2.4%	9	4.6%
External causes of morbidity and mortality	0	0%	0	0%	4	4.1%	31	2.3%	69	2.7%	3	1.5%
Total	0	0%	4	100%	98	100%	1352	100%	2517	100%	196	100%
