# Supplementary material for: Cause of death in patients with poststroke epilepsy: Results from a nationwide cohort study
Source: PLoS One. 2017 Apr 5;12(4):e0174659. doi: 10.1371/journal.pone.0174659 (PMC5381780; doi:10.1371/journal.pone.0174659)
Supplement: S3 Table — (PDF) [file pone.0174659.s003.pdf]

Table S3 Death from suicide, drowning and transport accidents in patients with PSE.

	Poststroke epilepsy (4167 deaths)	
	n	%
Drowning (V90, V92 and T75.1)	0	0%
Transport accidents (V01-V99)	2	0%
Self harm/suicide (X60-X84)	8	0.2%
